# The ins and outs of muscle stem cell aging

**DOI:** 10.1186/s13395-016-0072-z

**Published:** 2016-01-18

**Authors:** Andrew S. Brack, Pura Muñoz-Cánoves

**Affiliations:** 1 Department of Orthopaedic Surgery, Eli and Edythe Broad Center of Stem Cell Research and Regeneration Medicine, University of California San Francisco, 35 Medical Way, San Francisco, CA 94143 USA; 2 Department of Experimental and Health Sciences, Pompeu Fabra University, ICREA and Ciberned, Dr. Aiguader, 88, E-08003, Barcelona, Spain

## Abstract

Skeletal muscle has a remarkable capacity to regenerate by virtue of its resident stem cells (satellite cells). This capacity declines with aging, although whether this is due to extrinsic changes in the environment and/or to cell-intrinsic mechanisms associated to aging has been a matter of intense debate. Furthermore, while some groups support that satellite cell aging is reversible by a youthful environment, others support cell-autonomous irreversible changes, even in the presence of youthful factors. Indeed, whereas the parabiosis paradigm has unveiled the environment as responsible for the satellite cell functional decline, satellite cell transplantation studies support cell-intrinsic deficits with aging. In this review, we try to shed light on the potential causes underlying these discrepancies. We propose that the experimental paradigm used to interrogate intrinsic and extrinsic regulation of stem cell function may be a part of the problem. The assays deployed are not equivalent and may overburden specific cellular regulatory processes and thus probe different aspects of satellite cell properties. Finally, distinct subsets of satellite cells may be under different modes of molecular control and mobilized preferentially in one paradigm than in the other. A better understanding of how satellite cells molecularly adapt during aging and their context-dependent deployment during injury and transplantation will lead to the development of efficacious compensating strategies that maintain stem cell fitness and tissue homeostasis throughout life.

## Background

Stem cells are essential for the maintenance and repair of many adult tissues during normal physiology or in response to damage. Operationally defined, stem cells produce daughter cells that differentiate to repair damaged tissue and self-renew to repopulate the stem cell pool. Although long lived, tissue resident stem cells do not retain their fitness and function indefinitely. The unique requirement of tissue resident stem cells to maintain themselves and form new specialized cells may explain why their decline has a greater detrimental impact than that of other cell types on tissue regeneration. Across different stem cell compartments, age-dependent changes that cause stem cell dysfunction are multifactorial, encompassing, systemic, local, and intrinsic factors [1].

Adult stem cells have tissue-specific properties, related to the tissue they serve, such as distinct rates of turnover and specialized differentiation programs. Yet, they also have many common characteristics. They transit between quiescence and activation stages, their chromatin adopts bivalent states to facilitate rapid differentiation of self-renewal, they are capable of undergoing symmetric and asymmetric divisions, their metabolism is specialized to adapt to their particular needs, and they are located within microenvironments which influence their functions [2, 3]. These specific and common traits intertwine with general aging mechanisms resulting in distinct phenotypes over time.

During aging, many tissues undergo changes in stem cell number and function that impact tissue homeostasis. Optimal stem cell function necessitates appropriate extrinsic support from the local microenvironment (niche) and systemic environment (circulation). Hence, aging of the stem cell local and systemic environment is pertinent to stem cell demise. Since the original demonstration using parabiosis that muscle repair was under the control of soluble factors present in serum, alterations in the composition of the systemic environment has been the prevailing model to explain defects in skeletal muscle repair during aging [4, 5]. With aging, it has also been shown that niche cells no longer provide appropriate growth factor support, thus altering their behavior. Inflammation, which increases in the aging circulation and niche, also impacts negatively stem cell functions [6–8].

Satellite cells constitute the principal stem cell pool of adult skeletal muscle. Genetic ablation studies and transplantation studies together confirm that Pax7+ satellite cells are sufficient and required for adult muscle repair [9–11]. In response to muscle damage, satellite cells transition from their normally quiescent state, enter the cell cycle, and expand and differentiate (exit the cell cycle) to form new muscle fibers and regenerate the injured muscle tissue [12]. In aged mice, muscle repair is blunted in a large part due to satellite cell dysfunction [13–18]. However, stem cell decline does not contribute relevantly to the age-related reduction of myofiber size (sarcopenia) in the absence of muscle damage [19].

Unlike other types of stem cells, such as hematopoietic stem cells, not only the function but also the number of satellite cells declines with aging [13, 14, 20]. In aged muscle, the number of stem cells can become limiting for regenerative capacity [13]. It is likely that there exists a quorum of muscle stem cells to effectively repair muscle, and the number will differ depending on the fitness of the cells and the environmental support.

At variance with other types of stem cells, information regarding the molecular pathways promoting aging of satellite cells is abundant. Parabiosis and whole muscle grafting experiments reveal that the mechanisms controlling the regeneration capacity of stem cells may be principally ascribed to changes in the aging environment [4, 21–23]. However, more recent data from studies using transplantation of purified satellite cells demonstrates that stem cell dysfunction during aging has a cell-intrinsic component [13–16].

In this review, we discuss what is known about the principal stem cells of skeletal muscle, focusing on the control of stem cell function from the extrinsic environment and intrinsic regulation. We specifically consider what degree of stem cell dysfunction can be ascribed to the age-associated changes in the environment, cell-autonomous changes, or both, what makes satellite cells more or less susceptible to aging; and whether this can shed light on potential rejuvenating strategies.

### Muscle stem cell function and the aging environment

The influence of the extrinsic environment on the efficiency of the regeneration process arouse from whole muscle transplantation experiments and parabiosis. Transplantation of whole muscle grafts of young and old mice showed that the regenerative ability of young and old muscles depended mainly on the age of the host, as young hosts allowed successful engraftment of both young and old muscles while both graft types failed to regenerate in old rats [24]. However, further studies showed delayed regeneration in old muscle grafts even in a youthful environment [7, 8]. This was ascribed to the altered inflammatory cell function at old age [25]. In agreement with these results, studies by Lee et al. [26], using different paradigms of muscle regeneration, support a delay rather than impairment in muscle regeneration at old age. More recent studies have shown, however, that whole muscles from geriatric mice (28 months of age or older) grafted onto young recipient muscles maintain a regenerative defect [14]. In support, Chakkalakal et al. compared muscle repair 30 days after BaCl_2_-induced muscle injury and observed that muscle fiber size did not return back to pre-injured levels in aged mice [13]. Possibly, the differences in the extent of regeneration among these studies might be due to the distinct ages, gender of mice, and models of muscle injury employed. It is noteworthy that even if repair of muscle is delayed rather than impaired, the rate at which an aged human tissue recovers after injury or surgery will have significant ramifications for their health and general well-being.

Parabiosis has been used to test the effects of circulatory or systemic factors of one animal on the other for over 100 years [5]. Anastomosis of the circulatory system between adult and old mice provided definite evidence that the effects of aging on muscle regeneration can be modulated by the age of the systemic environment [4]. Based on short-term injury-repair models, youthful factors accelerated muscle repair, and aging factors delayed repair [4]. In vitro cell culture experiments supported this observation at the level of the satellite cell and their progeny [4, 27]. This seminal work paved the path to the discovery of circulating factors as regulators of satellite cell aging. Numerous studies have now shown that aged muscle repair can be augmented by modulation of many signaling pathways including RTK/ERK, Notch, Wnt, TGFβ, and hormones, such as oxytocin, in vivo [23, 27–30]. Importantly, neutralization of Wnt and TGFβ signaling in old mice restored efficient muscle repair. It is worth noting that both signaling pathways antagonize Notch signaling, which is critical for satellite cell activation, proliferation, and self-renewal [23, 27, 31]. In addition, treatment of aged mice with the reproductive hormone oxytocin promotes muscle regeneration [28]. In sum, targeting cell-extrinsic regulators in old mice, via parabiosis (or exposure to factors in young serum in cell culture), via inhibition of Wnt and TGFβ signaling, or oxytocin administration, significantly rescues defective satellite cell-mediated muscle regeneration.

Recently, the assessment of the expression and role of a circulating factor, growth differentiation factor 11 (GDF11), has provided conflicting results [32, 33]. The results from the first study implied that circulating GDF11 levels decline with aging and showed that the treatment of old mice with recombinant GDF11 improved muscle regeneration after injury [33]. A subsequent study showed that GDF11 levels do not decline with aging and that administration of recombinant GDF11 protein does not affect repair of old muscle [32]. The reasons for the discrepancies are discussed elsewhere [34].

Overall, these findings demonstrate reversibility of satellite cell dysfunction in old animals through restoration by, or mimicking of, a young extrinsic environment. Due to the nature of the muscle injury assay, and the influence of many cell types to its outcome, it is not possible to determine if these stimulatory effects are acting at the level of the stem cell, myogenic progenitors, or different cell types. Indeed, direct evidence for non-stem cell-mediated control of muscle repair comes from studies investigating inflammation. A transient blood-mediated inflammatory response to muscle injury is key for successful regeneration [35, 36]. Paradoxically, organismal inflammation increases during aging [37]. Yet, the initial inflammatory response to muscle injury in old mice is delayed [7]. This, together with the increased production of osteopontin by old macrophages infiltrating injured muscle, could contribute to the poor regenerative outcome of aged muscle [30]. Indeed, neutralization of osteopontin rejuvenated the behavior of old satellite cells in vivo and in vitro, reinforcing the idea that age-related inflammatory responses become counterproductive for muscle regeneration. Furthermore, recent studies have shown a detrimental role for IL-6-Jak-Stat3 signaling [17, 18]. Thus, while transient inflammation in response to muscle injury is required for efficient regeneration, chronic elevation may be deleterious [17, 18]. Finally, a recent report showed that the increased levels of TGFβ in aged mice promoted inflammation, rather than exerting its canonical role in attenuating immune responses, and hence inhibition of TGFβ signaling improved regeneration [31]. Interestingly, increased levels of TGFβ have been recently shown to promote survival of fibro-adipogenic progenitors (FAPs), a muscle-resident mesenchymal stomal cell population known to crosstalk with satellite cells during acute muscle regeneration in young mice [38]. Therefore, increased TGFβ in aged environment may have deleterious functions on satellite cells through an increase in pro-fibrotic muscle-resident stem cell types.

### Muscle stem cell aging at the cell-autonomous level

In more recent years, the direct assessment of muscle stem cell potential via transplantation has become more commonplace. Stem cell transplantation was pioneered by Till and Mcullough 55 years ago [39, 40]. Today it is considered the gold standard assay to examine stem cell potential. A cell possessing stem cell properties will be able to proliferate and self-renew to repopulate the stem cell compartment and produce differentiated daughter cells in a viable recipient tissue. The standard transplantation approach involves the enrichment/purification of stem cells within a tissue by antibody detection against cell surface receptors, or fluorescent reporters of cell-specific gene expression. It is imperative that the donor stem cell is indelibly marked to distinguish the donor from the recipient.

Injection of tractable adult satellite cells as a population, and at the single cell level, has provided definite evidence for their stem cell status, based on the ability to proliferate, differentiate (fuse to recipient pre-injured muscle), and self-renew (reoccupy the niche and express satellite cell markers). Comparisons between purified satellite cells from adult and aged mice transplanted into pre-injured adult muscle show a substantial decline in the number of aged satellite cells that repopulate the niche and contribute to muscle fiber differentiation (Table 1). In agreement, engraftment of single muscle fibers (with their complement of satellite cells) from adult and aged mice into recipient adult muscle demonstrated an age-dependent decline in satellite cell engraftment and niche repopulation [15]. In contrast, Collins et al. demonstrated that satellite cell niche repopulation was comparable between adult and aged engrafted muscle fibers [41]. While different methodologies have been employed, which hinder direct comparison, on balance, it is clear that aged satellite cells are less capable of niche repopulation and differentiation under the context of transplantation into an adult host. This is consistent with numerous earlier studies that showed aged satellite cells and their progeny were less capable of proliferating and differentiating in ‘optimized’ growth conditions in vitro [12, 42, 43].

**Table 1:** Reports of age-related changes in muscle stem cell function based on purified satellite cell and single muscle fiber transplantation assays

Age of donor	SC or single fiber transplant	Cell number and purification	Genotype and age of recipient	IR host	Muscle injury	Method of detection	Analysis timepoint	Change in SC function (aged relative to adult)	Ref.
4 vs 24 m	SC	2000 Lin-VCAM+, INT-A7	4 m C57Bl6	No	BaCl_2_	H2B-GFP marked nuclei per muscle	30 dPI	SC and myofiber: 40 % decline	[13]
5, 22, 30 m	SC	10,000 FACS: Lin-CD31+/INT-A7	4 m NOD/SCID	No	CTX	Lentiviral GFP	21 dPI	Myofiber: 70 % decline	[14]
2 vs 24 m	SC	10–100 (limiting dilution series) FACS	2 m NOD/SCID	Yes	CTX	GFP/Luc or Myf5lacZ	30 d/60 dPI	100 SCs: not different. 10 SCs: 30 % decline in SC	[16]
3 vs 18 m	SC	10,000 Pax7-Zs-green	2–4 m mdx	No	Natural turnover + CTX	Pax-Zs-green and dystrophin antibody	21 dPI	SC: 70 % decline; myofiber: 50 % decline	[17]
4 vs 26 m	Single fiber	5–7 muscle fibers	4 m C57Bl6	No	BaCl_2_	SC: β-actin GFP/Pax7+	30 d PI	60 % decline SC repopulation	[15]
1–2 vs 22–30 m	Single fiber	1 muscle fiber	1 m mdx. NOD/SCID	Yes	Natural turnove + repeated CTX	Myonuclei :3F-nlacZ-2E. SC: Myf5lacZ	30 d/40 dPI	SC and myofiber: not different	[41]

The results highlight the different approaches that have been employed to study the effect of age on satellite cell engraftment, self-renewal, and differentiation using purified satellite cell and single muscle fiber transplantation assays

*Abbreviations*: *m* month, *Lin* ^−^ Sca1^−^/CD31^−^/Cd45^−^, *NOD*/*SCID* non-obese diabetic/severe combined immune deficiency, *Mdx* mouse model carrying naturally occurring mutation of dystrophin gene, *IR* irradiation, *CTX* cardiotoxin, *BaCl*_*2*_ barium chloride, *dPI* days post injury, *GFP/LucZ* ubiquitous readout, *Myf5lacZ* lacZ reporter of *myf5* locus, *Ref.* reference

Over the past few years, it has become apparent that functional heterogeneity exists within the satellite cell pool [13, 44–46]. Therefore, any functional decline in the aged satellite cell pool under transplantation conditions might exist through a relative loss of competent cells rather than the uniform loss of function across the total pool. Using a TetO-H2B-GFP reporter to quantify proliferative output, it is apparent that based on the retention (label retaining cells; LRCs) or loss of GFP label (non-LRCs; nLRCs), the adult satellite cell pool is composed of cells that undergo distinct proliferative histories during postnatal growth and regeneration [13]. Examined under transplantation, LRCs are able to self-renew and differentiate, and nLRC are limited to differentiation. Therefore, LRCs act as stem cells and nLRCs as committed progenitors. It is estimated that there are ~65 % fewer LRCs in aged versus adult muscle [13]. Importantly, the remaining LRCs in aged muscle retain a similar level of tissue engraftment and contribution as adult LRCs, i.e., they are age-resistant. Cosgrove examined satellite cell function in transplantation assays under a limiting dilution series, whereby satellite cells ranging from 10 to 100 were injected into a pre-irradiated, pre-injured adult muscle [16]. It was calculated that aged muscle contains ~65 % fewer functional stem cells than adult muscle. Together, these data argue strongly that the functional decline observed in the aged satellite cell pool is due to a decrement in the number of functional stem cells.

### Parabiosis versus transplantation paradigms to study muscle stem cell aging

The functional deficit of aged satellite cells engrafted in a young host in its strictest sense has been used to conclude that there is a non-reversible component to stem cell aging. However, parabiotic conditions provided only a partial recovery of muscle repair in aged mice exposed to a young systemic environment. One could consider this due to a partial dilution of aged blood; however, it could also be due to other factors outside the systemic environment, including the niche or intrinsic defects. To date, a direct comparison of aged satellite cells after engraftment in adult and aged recipients is lacking. In addition, using heterochronic parabionts to serve as the source of donor satellite cells or recipient muscle for transplantation experiments has not been examined. For example, if the functional deficit of aged satellite cells (versus adult satellite cells) engrafted into an aged host was twice that observed in a young host, the results from parabiosis, transplantation, and in vitro studies would be in general agreement: that alterations in stem cell function during aging is a product of intrinsic and extrinsic deregulation. Alternatively, if results from transplantation studies do not support an extrinsic component, in contrast to endogenous muscle cells after injury, this would highlight the context dependence of the assays employed. Another factor that limits direct comparison is the age of the mice examined. Parabiosis studies have classically been carried out on 18-month-old mice, whereas transplantation studies have predominantly used mice that are 20 months and older. As age-dependent phenotypes are progressive, this may impact the rejuvenation potential of satellite cells. Another distinction is the length of recovery after injury between the two types of studies. Acute repair processes have been studied predominantly after parabiosis, and longer recovery periods assessed in transplantation studies. Identifying the long-term injury response on parabionts would help to resolve this potential discrepancy. Finally, we cannot discard that the genotype and age of the recipient mice in cell engraftment experiments might also influence the regenerative outcome. Indeed, aged mice experience chronic inflammation, yet the inflammatory response in whole muscle grafts is less efficient with age, and this appears to impact new myofiber formation [8]. Consistent with the influence of the inflammatory status of the recipient mice, it is tempting to propose that the exacerbated inflammation of *mdx* mice or the blunted immune response of immune-compromised mice will exert opposite, albeit impactful effects on cell engraftment.

Resolving the relative contribution of intrinsic and extrinsic control on stem cell function is critical, but it should also be considered that the assays deployed for determination are not equivalent and may overburden specific cellular regulatory processes and thus probe different aspects of satellite cell properties. The cellular experience of the stem cell pool is likely very different if activated by injury while in their resident niche, compared to the physical manipulations that take place to extract, purify, and inject satellite cells into a foreign muscle. It is likely that the isolation of satellite cells followed by their transplantation (sometimes with a prior cell culturing period) may provoke cellular changes (epigenetic, transcriptional, or other) that influence satellite cell output and tissue contribution. On the other hand, parabiosis procedure-associated complications like limitation on mice mobility, chronic inflammation (which perturbs the environment), or hypoglycemia, may account for some of the observed effects [47].

Intriguingly, in other stem cell systems, different stem cell subsets can dominate tissue contribution depending on the assay employed. Using genetic bar coding, which allowed individual hematopoietic stem cells (HSC) clones to be tracked over time, the Camargo lab found that HSC contribution during transplantation was derived from substantial expansion of rare clones [48]. In contrast, normal blood production was derived from more modest expansion of multiple clones. If this feature of stem cell diversity is conserved, one could hypothesize that a rare population of satellite cells are equipped to successfully engraft, self-renew, and differentiate under the setting of transplantation, whereas a larger fraction of the satellite cell population is equipped for robust tissue contribution during endogenous muscle repair. Hall et al. [49] examined the fate of transplanted adult satellite cells as the host aged. Analysis of aged muscle 21 months after initial satellite cell engraftment showed that that engrafted population persisted and increased their relative contribution over the host satellite cell population over time. This data suggests that the satellite cell pool contains age-resistant highly engraftable subsets. One can speculate that the properties that endow transplantation capacity and render age-resistance may overlap. In the future, it will be important to define the contribution and regulation of satellite cell subsets in distinct contexts.

### Loss of satellite cell functions during aging

#### Cell-autonomous alterations

The functional deficit of satellite cells as a feature of aging examined under transplantation suggests a strong cell-intrinsic and irreversible component that could include genomic instability, DNA and oxidative damage, and alterations in mitochondrial function. Indeed, satellite cells have an increased resistance to DNA damage and capacity to repair DNA lesions than their committed progeny [50]. Satellite cells isolated from aged muscles show an increased number of foci containing the phosphorylated form of histone H2AX (pH2AX), a marker of DNA damage [14, 33]. In addition, the decline in antioxidant capacity of satellite cells with aging [51] likely impacts their genomic integrity, suggesting a relevant function of DNA damage repairing mechanisms. Consistent with this, mice deficient for Ku80, a subunit in the non-homologous end-joining pathway (NHEJ), show an accelerated aging phenotype in skeletal muscle [52]. Comparative gene expression studies from quiescent and activated satellite cells from young and old mice and humans have uncovered transcriptomic changes related with decreased antioxidant activity and altered expression of myogenic differentiation-specific genes and genes related to protein folding [41, 53–59], which could be caused by global epigenetic alterations in young and old satellite cells [58].

At the level of single genes, adult satellite cells epigenetically repress the INK4a locus that encodes p16^INK4a^ (a cell cycle inhibitor and marker of senescence) via ubiquitination of histone H2A, this repression is relieved in aged cells [14]. Furthermore, increasingly permissive chromatin has been detected at the p16^INK4A^ and p21^CIP1^ loci, provoked by alterations in H3K4me3 and H3K27me3 chromatin marks, in aging satellite cells after injury [28].

Despite the always attractive possibility for rejuvenation as indicated by parabiosis experiments, satellite cell aging proceeds to a limiting point where it essentially becomes irreversible, particularly at the late geriatric age [14], suggesting further intrinsic changes in geriatric cells. In the quiescent state, geriatric cells express p16^INK4a^, which causes loss of the G0 reversible quiescent state. In response to regenerative pressure by injury, p16^INK4a^ caused the de-phosphorylation of the retinoblastoma protein (Rb) and repression of E2F target genes in proliferation-promoting conditions, which accelerates entry into full senescence [14]. Regardless of the more or less general senescence occurrence, elimination of senescent cells through inducible targeting of p16^INK4a^ has been shown to delay aging in several tissues of BubR1 progeroid mice, including skeletal muscle [60]. It remains unresolved whether the phenotypic improvement after p16^INK4a^ knockdown or ablation of p16^INK4a^-expressing cells is restricted to senescence modulation exclusively.

Whether the pre-senescent state of satellite cells from geriatric mice is restricted to a subset or a feature of most satellite cells is not known. However, it is tempting to propose that, as for satellite cell apoptosis, senescence might be an alternative way to restrict unfit satellite cells of aged mice from undergoing myogenesis. Supporting the notion that p16^INK4a^-induced senescence can contribute to loss of satellite cells, mice overexpressing p16^INK4a^, due to genetic loss of the polycomb repressor complex 1 (PRC1) protein Bmi1 [14, 61], or the enzymatic subunit of the polycomb-repressive complex 2 (PRC2), Ezh2 [62], showed reduced numbers of satellite cells. Progeric mice displaying premature aging also showed a depletion of the satellite cell pool, increased p16^INK4a^ expression in the resting state, and pro-senescence phenotypes [14]. Interestingly, a recent report demonstrated cellular senescence in the process of adult skeletal muscle regeneration [63], suggesting that this cellular fate is not restricted to aging muscle.

### Extrinsically driven satellite cell alterations

While intrinsic molecular effectors determine cell functionality, it is less clear whether they are autonomously driven or a consequence of altered extrinsic factors. The adult satellite cell pool is functionally heterogeneous, with subsets enriched for stem cell potential under transplantation conditions [13, 44, 45, 64]. During aging, there is a loss of muscle stem cells, that is associated with a decrease in *Spry1* (encodes Sprouty1), an inhibitor of FGF signaling and upregulation of FGF2 in the niche. Genetic elimination of *Spry1* in satellite cells enforced FGF signaling, which resulted in loss of quiescence and subsequent reduction in the satellite cell number. *Spry1* elimination during adult muscle repair led to sustained activation of the ERK pathway, which impaired self-renewal and caused apoptosis in a subset of satellite cells [65]. Together, these findings reinforce an FGF-feedback regulatory mechanism for stem cell viability [13]. The critical role of *Spry1* in muscle stem cell regulation is not restricted to murine studies. Myogenic progenitors from adult and aged human biopsies show increased DNA methylation at the *Spry1* locus coupled with a loss of ‘reserve’ cells to return back to a quiescent state [66]. Demethylation of the aged cells improved self-renewal of the quiescent pool. This suggests that there may be cell-autonomous regulation of pathways classically considered extrinsically driven. It would be interesting to examine whether niche-derived growth factors can shape the epigenetic landscape of the stem cell.

The normal asymmetric divisions that produce one stem and one committed progenitor in adult satellite cells are disrupted in aged satellite cells via chronic elevation of p38αβ MAPK signaling and desensitized FGFR1 activity. Instead, aged satellite cells preferentially undergo symmetric divisions that produce two committed daughters. This limits the expansion of aged satellite cells, favoring their permanent cell cycle exit [15, 16]. While it is not clear what drives p38αβ MAPK in aged satellite cells, niche-derived FGF2 is a feasible candidate. Bernet and colleagues propose that elevated FGF2 is a compensation strategy to remedy the loss of FGFR1 signaling [15]. Alternatively, reduced FGFR1 sensitivity may be a consequence of ligand-induced receptor internalization [67]. In conclusion, the FGF2-p38αβ MAPK pathway has detrimental consequences on the intrinsic regenerative functions, including the restoration of the quiescent satellite cell pool through self-renewal.

Similarly, as indicated before, an increasingly active IL-6-JAK/STAT3 pathway in satellite cells with age appears to reduce their regenerative potential by limiting their proliferative capacity and affecting their symmetric versus asymmetric polarization [17, 18]. This may be linked to the higher levels of inflammatory cytokines found in aging organisms [68].

### Stress pathways in aged satellite cells

It is widely appreciated that freshly isolated satellite cells from aged mice have a predisposition for apoptosis and senescence when placed in culture conditions [13, 14, 16, 41]. This suggests that aged satellite cells may be less resilient in the face of exogenous stress and undergo molecular changes that limit functional output.

Recent data from multiple groups have shown a robust improvement of aged satellite cells under transplantation by various intervention strategies. Interestingly, adult satellite cells are less responsive to the same interventions. These recently identified age-related intrinsic alterations include a combination of signaling pathways associated with cellular stress, such as p38 MAPK, JAK-STAT3, and p16^INK4a^. Elevation of stress pathways beyond a certain threshold can promote cellular arrest and predispose to senescence or apoptosis fates, either by inducing mitochondrial dysfunction, increasing reactive oxygen species (ROS), or DNA damage (reviewed in [69–73]). The cumulative alterations of stress-related pathways with aging, and their hyperactivation during experimental cell transplantation procedures, limit the expansive capacity and engraftment of old satellite cells and impair formation of new myofibers. Importantly, caloric restriction as a means to decrease metabolic stress improved engraftment of adult satellite cells and expansion of adult and aged satellite cells in cell culture assays [74]. Importantly, stress-dependent control of stem cell function is likely conserved across species [75]. In contrast to adult human satellite cells that lose engraftment after expansion in culture, human satellite cells grown in the presence of a p38 MAPK inhibitor can proliferate in vitro and retain subsequent engraftment potential in transplantation assay [75]. Therefore, transplantation success will likely depend on the ability to adapt and survive the stress of engraftment. Because aged satellite cells may be less resilient, modulation of stress pathways could provide a robust intervention for their functional rejuvenation.

The satellite cell pool becomes more homogeneous with age, with reduced representation of cells having high regenerative potential, and increased proportion of cells with reduced clonal proliferation capacity [13]. Thus, it is likely that the functional improvement of old satellite cells by biochemical or genetic strategies in transplantation experiments results from the proliferative amplification of a minor subset of highly regenerative cells. In any case, aged satellite cells seem susceptible to the high levels of stress induced by transplantation procedures, and only attenuation of the stress (i.e., by reducing p38 MAPK, JAK-STAT signaling, or p16^INK4a^-induced senescence) can allow their functional rejuvenation upon engraftment.

## Conclusions

Parabiotic studies have demonstrated extrinsic components, whereas transplantation assays have confirmed intrinsic components that cause age-dependent muscle stem cell decline. At first glance, the results based on satellite cell transplantation appear to be in conflict with those from parabiosis. The main purpose of this review has been to highlight the potential causes of discrepancy among the different papers and discuss how these differences teach us new facets about muscle stem cell biology and aging. We believe that the procedure chosen to assess the capacity of the young environment to rejuvenate aged satellite cells greatly accounts for the differences. We propose that the isolation of satellite cells and their subsequent transplantation may exacerbate intrinsic changes that importantly alter their functions. Thus, stem cell potency is greatly influenced by their capacity to overcome the stress of engraftment. From this viewpoint, old satellite cells would be less resistant to this stress. Importantly, strategies that modulate stress pathways in old satellite cells constitute a robust method for functional rejuvenation. Noteworthy, acute inflammation and stress, induced by the surgical joining of two animals in parabiosis experiments, might cause systemic perturbations that could affect satellite cell functions in yet unpredictable ways. In addition, the choice of genotype and age of the recipient mice for engraftment assays might also influence the regenerative outcome.

Another issue that may contribute to the apparently discrepant results between parabiosis and transplantation experiments relies on the molecular and functional diversity within the satellite cell population. Rejuvenation of aged satellite cells, in the context of transplantation, might derive from the augmented expansion of a small subpopulation of the fitter and more stress-resistant stem cells. Whether distinct satellite cell subsets are mobilized preferentially in the context of repair and transplantation or are differentially sensitive to intrinsic and extrinsic stressors warrants testing. A greater comprehension on the causative factors that drive satellite cell dysfunction during aging will lead to strategies that promote tissue rejuvenation and a beneficial impact on the quality of life of the elderly.
